# Patient-related risk factors for unplanned 30-day readmission following total knee arthroplasty: a protocol for a systematic review and meta-analysis

**DOI:** 10.1186/s13643-019-1140-3

**Published:** 2019-08-22

**Authors:** Daniel Gould, Michelle Dowsey, Tim Spelman, Imkyeong Jo, Wassif Kabir, Jason Trieu, Peter Choong

**Affiliations:** 1 University of Melbourne Department of Surgery at St. Vincent’s Hospital Melbourne, Level 2 Clinical Sciences Building, 29 Regent Street, Fitzroy, 3065 Australia; 2 Department of Othopaedics at St. Vincent’s Hospital Melbourne, Level 3 Daly Wing, 35 Victoria Parade, Fitzroy, 3065 Australia

**Keywords:** Risk factors, 30-day readmission, Hospital readmission, Unplanned readmission, Total knee replacement, Total knee arthroplasty, Patient characteristics

## Abstract

**Background:**

Osteoarthritis is a debilitating condition as well as a growing global health problem, and total knee arthroplasty is an effective treatment for advanced stages of disease. Unplanned 30-day hospital readmission is an indicator of complications, which is a significant financial burden on healthcare systems. The objective is to perform a systematic review of patient-related factors associated with unplanned 30-day readmission following total knee arthroplasty. This information will inform future strategies to improve health outcomes after knee arthroplasty surgery.

**Methods:**

MEDLINE and EMBASE will be systematically searched using a comprehensive search strategy. Studies of higher quality than case series will be included, in order to optimise the quality of the findings of this review. We will include studies reporting on patient-related risk factors for unplanned 30-day readmission following primary or revision total knee arthroplasty for any indication. Case series will be excluded, as will studies reporting exclusively on intraoperative, clinician, hospital, and health system risk factors. The reference lists of selected papers will then be screened for any additional literature. Two reviewers will independently apply stringent eligibility criteria to titles, abstracts, and full texts of studies identified in the literature search. They will then extract data from the final list of selected papers according to an agreed-upon taxonomy and vocabulary of the data to be extracted. Assessment of risk of bias and quality of evidence will then take place. Finally, the effect size of each identified risk factor will be determined; meta-analysis will be performed where adequate data is available.

**Discussion:**

The findings of this review and subsequent meta-analysis will aid clinicians as they seek to understand the risk factors for 30-day readmission following total knee arthroplasty. Clinicians and patients will be able to use this information to align expectations of the postoperative course, which will enhance the recovery process, and aid in the development of strategies to mitigate identified risks. Another purpose of this review is to assist policy-makers in developing quality indicators for care and provide insights into the drivers of health costs.

**Systematic review registration:**

PROSPERO CRD42019118154.

**Electronic supplementary material:**

The online version of this article (10.1186/s13643-019-1140-3) contains supplementary material, which is available to authorized users.

## Background

### Total knee arthroplasty—effectiveness and trends over time

Total knee arthroplasty (TKA) is a highly effective treatment for advanced stages of knee osteoarthritis (OA). The procedure improves health-related quality of life [1] and TKA has good long-term survivorship, with a cumulative percent revision of just 5.3% at 10 years in Australia [2]. The incidence and prevalence of OA are increasing [3, 4], and there has been a corresponding increase in the utilisation of TKA [2, 5, 6] with an annual increase in procedures of over 123% since 2003 in Australia. The most recent census reports over 55,000 primary TKA procedures in 2017 [2]. This trend of increased TKA utilisation is expected to continue both in Australia [7] and internationally [8, 9]. In the USA alone, a 673% increase in the demand for TKA is expected for the 25 years up to 2030 [9]. Accompanying the increased utilisation of TKA is a change in patient demographics [8, 10, 11], including increasing age, BMI, and physical activity demands. In this changing population of TKA recipients, an understanding of the risk factors for 30-day readmission following joint arthroplasty will help clinicians to better understand the postoperative course of their patients. Knowing the potential risks which require specific mitigation and how to identify patients with the highest risk of readmission, will improve the effectiveness and efficiency of care.

### The importance of 30-day readmission

Readmission within 30 days of index procedure is a complex relationship between the general health and physiology of the patient and the morbidity of the surgery [12, 13]. How well patients are managed by institutional processes (medical and logistic) may influence the rate of readmission, with hospitals being held accountable for high 30-day readmission rates [14] and this parameter being perceived as an indicator of the quality of care [15–18]. While the use of 30-day readmission rate as a quality measure has been called into question [19, 20], reducing it is beneficial to both the patient and institution due in part to patient dissatisfaction and the significant financial burden associated with 30-day readmissions following TKA [21–24], respectively. In addition to the financial implications of hospital readmissions, it is important for patients receiving TKA to have realistic expectations regarding their postoperative course in order to increase the likelihood of achieving a satisfactory outcome [25]. This review will assist clinicians to predict an individual patient’s risk of readmission, so that patients may provide more informed consent for surgery and healthcare providers may develop better strategies to mitigate this risk. Better management of the post-operative course is likely to impact positively on the patient’s expectation and satisfaction [26–30].

### Common reasons for 30-day readmission post-TKA

Among the prominent indications for 30-day readmission following TKA are surgical site infection (SSI) and cardiovascular event [31], and each of these has been shown to increase costs in the first 30 days following surgery [24]. Risk factors for early SSI following TKA include hypertension, perioperative blood transfusion, use of oral corticosteroids, elevated serum neutrophil count, and use of warfarin or rivaroxaban for venous thromboembolism (VTE) prophylaxis [32]. Although focusing mostly on 90-day complications, Singh et al. [33] reported that risk factors for postoperative cardiac or thromboembolic events following TKA included American Society of Anesthesiologists (ASA) class III–IV, male gender, and higher Charlson index score. The metabolic syndrome is also correlated with cardiovascular complications following total joint arthroplasty [34], although it is unclear how relevant this is to the 30 days immediately following operation.

### Short length of stay and outpatient TKA

There is a prominent trend towards reduced hospital length of stay (LOS) in TKA [35]. Extending on this trend, there has also been increased utilisation of outpatient TKA [35–37]. Short LOS is correlated with increased 30-day readmission rates [38] and also contributed to the overall increase in 30-day readmission rates between 1991 and 2010 in the USA [6]. Contrasting these findings, increased TKA 30-day readmission rate has not been correlated with TKA performed as an outpatient procedure in selected patients [39, 40].

### Previous systematic reviews—30-day readmission in orthopaedics linked to increased BMI, age, and ASA classification

To the best of our knowledge, only one systematic review has been published on 30-day readmission in orthopaedics—the 2015 systematic review by Bernatz et al. [41], which includes an analysis of risk factors for readmission in orthopaedic surgery. However, there has not been a systematic review published concerning the risk factors for 30-day readmission in TKA patients specifically. Some of the risk factors Bernatz et al. identified included age, increased body mass index (BMI), and ASA class greater than IV. Diabetes, male sex, and history of pulmonary disease were not found to increase the risk of readmission in orthopaedic patients overall.

The findings from some studies specifically investigating TKA patients have also reported increased 30-day readmission risk with older age [12, 42, 43], increased BMI (12—specifically, obesity), and high ASA class [42], but accompanying these concordant findings are important discrepancies in the available evidence focused on the TKA population. For example, some authors [44] did not find a correlation between increasing BMI and 30-day readmission, and Varacallo et al. [45] reported that age was not a significant risk factor. Furthermore, while Varacallo et al. [45] and Tayne et al. [46] found high ASA class to be a risk factor, this contrasted with the findings of others (Courtney et al. [40] and Sutton et al. [47]). It is important to note that Varacallo et al. [45] and Tayne et al. [46] reported on risk factors for readmission in a combined cohort of TKA and THA patients, so the findings may not be applicable specifically to the TKA population.

In summary, the body of evidence concerning risk factors for 30-day readmission in TKA patients exhibits discrepancies for prominent demographic factors such as age and BMI, and for ASA class which encompasses comorbidity burden.

### The rationale for this systematic review and meta-analysis

This systematic review will address gaps and contrasting findings in the current literature around patient risk factors for 30-day readmissions after TKA. Specifically, a lack of knowledge exists in the areas of demographic characteristics such as age and sex, as well as comorbidity burden and individual comorbidities. Our findings will inform newer healthcare strategies that will impact patients and healthcare delivery, specifically targeting patient satisfaction, clinical outcomes and cost. When combined with existing knowledge, this systematic review will provide evidence to arm prediction tools that may be used to identify high-risk patients. For modifiable risk factors such as BMI and patient general health, this review could help to shape practices that target risks of readmission by addressing known modifiable risk factors and carefully selecting patients for surgery. Patients can also prepare appropriately for their likely postoperative course given their individual risk of 30-day readmission.

## Objectives

The objectives of this review are to (1) identify patient-related characteristics which confer increased risk of unplanned 30-day readmission following TKA and (2) determine the effect size of the association between the identified risk factors and unplanned 30-day readmission. The systematic review will synthesise existing knowledge, while the meta-analysis will be used to determine the effect size of identified factors and resolve uncertainty when discrepancies arise between reports.

## Methods/design

### Eligibility criteria

- Population = TKA recipients
- Outcome variable = 30-day readmission to any institution, due to any cause
- Comparator = N/A
- Predictor variable = Patient risk factors
- Study type = Case series will be excluded. All other types of quantitative study design are eligible for inclusion, including retrospective and observational studies

There will be no restriction placed on the date of publication for inclusion in this review. We aim to capture all of the available evidence concerning patient-related risk factors for 30-day readmission in TKA.

These eligibility criteria were selected to answer the following question: which patient-related factors confer increased risk of unplanned 30-day readmission following total knee arthroplasty, and to what extent do these factors influence the risk?

### Reporting

Reporting will follow the PRISMA guidelines, according to the PRISMA-P checklist (Additional file 1).

### Information sources

The electronic bibliographic databases MEDLINE(Ovid), EMBASE(Ovid), and Cochrane Library will be searched. There will be no limit applied to the search strategy regarding publication period or the language of publication, although studies in languages other than English (LOE) will be excluded from the review and this will be discussed as a potential source of language bias in the ‘meta bias’ section. Additional studies will also be identified by searching the reference lists of the included studies.

The search will be repeated immediately prior to final analysis to ensure retrieval of additional research.

### Search strategy

Cochrane Library has been searched, using the MeSH term “Arthroplasty, Replacement, Knee” to ensure a Cochrane review has not been published on the topic of risk factors for 30-day readmission following TKA.

The full search strategy for the MEDLINE(Ovid) and EMBASE(Ovid) databases is included as Additional file 2.

### Data management

Retrieved records will be managed using EndNote software, with subgroups generated in order to maintain a clear record of inclusion/exclusion decisions. Extracted data will be managed in a spreadsheet available to reviewers.

### Study selection

Using agreed eligibility criteria pilot tested on a subset of abstracts identified in preliminary searches and then refined by a discussion between reviewers in accordance with PRISMA guidelines, two reviewers will independently screen titles and abstracts of identified citations. Discrepancies will be resolved by discussion between reviewers. The eligibility criteria will then be applied by the same reviewers to the full text of potentially eligible studies. Authors of potentially eligible studies lacking pertinent data will be contacted, via email, in order to obtain the required data—total number of TKA patients in the study, number of readmissions in the overall TKA cohort, number of TKA patients analysed for each reported patient-related factor, and the number of readmissions for each factor—for inclusion of the study. If the relevant data cannot be obtained, the study will be excluded.

Additional studies will then be identified by searching the reference lists of those articles included in the final list of papers identified following application of the finalised eligibility criteria.

When multiple reports from the same study cohort or database are identified, care will be taken to determine whether the follow-up periods and characteristics of the separate reports are the same. If this is the case, these reports will be treated as the same study but reference will be made to all of the publications [48]. These studies will also be compared in order to highlight any discrepancies in the findings, and authors of the studies will be contacted for clarification if such discrepancies are identified

### Data collection process

Two reviewers will independently extract data from each study using a standardised data extraction. The data extraction forms completed by each reviewer will be compared for consistency and accuracy. Inconsistencies will be resolved through discussion and consultation with the other authors. Authors will be contacted directly via email if papers are unobtainable, and if clarification is required regarding methods or results of included papers.

### Data items

The data extraction form will contain the following information:

- Details of the paper: first author, year of publication, name of the paper, publication journal, and study design (prospective or retrospective)
- Clinical setting: geographical location, sample size, and public/private/university-affiliated institution
- Characteristics of the patients, including demographics (such as age, sex, body mass index (BMI)) and comorbidities (such as diabetes, smoking status, and ASA classification)
- Results summary—required information for risk ratio (RR) calculation: total number of patients in the study; the total number of readmitted patients in the study; risk factors for unplanned 30-day readmission—total number of patients with each identified risk factor, and number of patients with the given risk factor who were readmitted

### Outcomes and prioritisation

Characteristics of the primary outcome: unplanned readmission to any institution within 30 days post-TKA. There is no secondary outcome of interest.

### Risk of bias assessment

Two reviewers will independently apply the following tools to assess the risk of bias in included studies: Cochrane risk-of-bias tool for randomised trials [49], Joanna Briggs Institute (JBI) checklists [50] for each other study type. We anticipate that we will mainly use the JBI critical appraisal tool designed for cohort studies since hospital medical record readmission data is readily available from multiple databases, and is therefore amenable to retrospective cohort study design. We may modify the relevant critical appraisal tool if there are elements which do not apply to the studies included in the review. Such modifications, with appropriate justification, will be documented accordingly. Results of the critical appraisal process will be presented in a summary table, separate to the data summary table, which will include all of the articles in the systematic review. Those articles selected for meta-analysis, based on adequate data for quantitative synthesis, which have a high risk of bias (‘exclude’ according to the JBI checklist) will be excluded as part of a sensitivity analysis [51, 52] to determine whether and to what extent the conclusions of the meta-analysis are influenced by the inclusion of these studies.

### Data synthesis and statistical analysis

Quantitative synthesis of the findings from the included studies is planned. The Cochran Q test and I^2^ statistic will be used to assess heterogeneity and will be addressed according to the approach outlined in the Cochrane Handbook [52]. Concretely, subgroup analyses will be carried out whenever significant heterogeneity is encountered, with random-effects meta-regression utilised only when there are ≥ 10 studies included in the given comparison.

If the heterogeneity is not resolved following this process, exclusion of outlying studies will be considered as part of a sensitivity analysis if there is a clear explanation for the conflicting results found upon rigorous review of the papers in question. The results of the meta-analyses both including and excluding the relevant studies will be included in the review and interpreted appropriately.

If there is considerable variation in results which cannot be accounted for then meta-analysis will not be conducted. Instead, results will be combined in a narrative synthesis with the results of included studies presented in forest plots (without pooled estimate) wherever applicable, namely, whenever two or more studies report on the same risk factor.

Otherwise, if deemed suitable, random-effects meta-analysis will be conducted using RevMan Software. Suitable studies will be those which report the number of TKA patients for each reported risk factor as well as the number of readmissions for that risk factor, such that the risk ratio (RR) can be calculated for the dichotomous primary outcome variable. RR was selected because it is more intuitive [53] than odds ratio (OR) and therefore easier to communicate. Furthermore, estimates of the 30-day readmission rate for TKA range from 3% [54] to 4.6% [42] and at an incidence of outcome below 10% the OR would be expected to provide a reasonable approximation of the RR [55]. When at least two studies [56, 57] report on the same risk factor, the RRs will be presented in a forest plot with pooled estimate and 95% confidence interval. For risk factors reported in only one study, the RR with 95% confidence interval will be reported in written form.

Care will be taken to ensure data will only be pooled from studies reporting the same variable in the same form—for example, studies which analysed age as a continuous variable will only be pooled with other studies which reported on age as a continuous variable and not to those which analysed age as a categorical variable. This will clarify the effect size of applicable data and resolve differences between studies.

### Meta-bias

Selective reporting within studies will be investigated as part of the data extraction process, during which the two reviewers will systematically check each paper to ensure all of the risk factors for 30-day readmission documented in the methods section are reported in the results, even in the event of a non-significant impact on readmission risk. The completed data extraction table will then be assessed by the reviewers to determine whether there are particular studies which did not address certain risk factors which were commonly reported in the majority of included studies. If this is the case, the pertinent studies will be investigated for possible reasons to explain this apparent lack of information and authors may be contacted to provide it [52].

Publication bias will be minimised by the use of a comprehensive search strategy which places no restriction on ‘grey literature’ [52, 58]—data from unpublished work will be identified and included as part of the meta-analysis. To reduce the risk of publication bias in included studies, where > 10 studies report the same risk factor a funnel plot will be generated and appropriate tests of asymmetry will be carried out in order to accurately interpret the findings [52, 59]. If evidence of small-study effects is detected, the implications of these effects may be considered in sensitivity analyses [52].

Although literature in LOE will be excluded, the number of such studies will be reported, and all available English language abstracts of LOE publications will be screened to determine their potential eligibility if they were to be considered for inclusion in the review. The findings will be documented and discussed as a potential source of language bias [52]; however, Morrison et al. found no evidence of a systematic bias from the use of language restrictions in systematic review-based meta-analyses in conventional medicine [60].

### Confidence in cumulative evidence

The Grades of Recommendation, Assessment, Development and Evaluation (GRADE) approach [52, 61] will be employed to generate a Summary of Findings (SoF) table according to the process developed by Rosenbaum et al. [62]—appropriate consideration will also be given to the presentation of findings from observational studies in SoF tables [63, 64], given the high proportion of retrospective cohort studies expected to be included in this review. SoF tables have been found to improve understanding and rapid retrieval of prominent findings from reviews [65]. As such, this will be a key component of the review in relation to its intended use as a reference to guide the decision-making process for policy-makers and clinicians [66].

## Discussion

The results of this systematic review will aid clinicians to identify which patients are more likely to be readmitted within 30 days of their TKA procedure, given their risk profile. It will also assist policy-makers in better understanding the patient-related factors contributing to increased risk of readmission, and this can be taken into account when using hospital readmission rates as an indicator of the quality of care; if some of the modifiable risk factors can be targeted, perhaps readmission rates could be reduced.

This review is restricted to patient risk factors; therefore, it is important that clinicians do not draw inferences about other types of risk factors, including intraoperative, clinician, hospital, and health system risk factors.

Potential issues which could occur in the conduct of this review include possible lack of randomised controlled trials due to the nature of the topic (i.e. patients are being compared for the same intervention. Cohort studies are therefore the best possible study design to investigate patient risk factors for 30-day readmission), significant proportion of duplicate publications owing to multiple studies using the same large-scale databases, and the possible exclusion of studies reporting readmission risk in a hip and knee arthroplasty population (total joint arthroplasty) for which TKA-specific data cannot be obtained from authors.

The review is potentially limited to a small degree by the exclusion of LOE literature.

## Additional files

Additional file 1: Completed PRISMA-P Checklist. (DOCX 33 kb)

Additional file 2: Search strategies (MEDLINE(Ovid) and EMBASE(Ovid)). (DOCX 7 kb)

## Data Availability

Additional file 1 is the completed PRISMA-P Checklist. Additional file 2 details the MEDLINE(Ovid) and EMBASE(Ovid) search strategies.

## Abbreviations

ASA: American Society of Anesthesiologists
BMI: body mass index
GRADE: Grades of Recommendation, Assessment, Development and Evaluation
JBI: Joanna Briggs Institute
LOE: Languages other than English
LOS: Length of stay
OA: Osteoarthritis
OR: Odds ratio
RR: Risk ratio
SoF: Summary of findings
SSI: Surgical site infection
TKA: Total knee arthroplasty
VTE: Venous thromboembolism
