# Supplementary material for: Patient-related risk factors for unplanned 30-day readmission following total knee arthroplasty: a protocol for a systematic review and meta-analysis
Source: Syst Rev. 2019 Aug 22;8:215. doi: 10.1186/s13643-019-1140-3 (PMC6706890; doi:10.1186/s13643-019-1140-3)
Supplement: Supplementary file 2 — Search strategies (MEDLINE(Ovid) and EMBASE(Ovid)). (DOCX 7 kb) [file 13643_2019_1140_MOESM2_ESM.docx]

| **MEDLINE (Ovid) Search Strategy** | |
| --- | --- |
| 1 | Exp *Arthroplasty, Replacement, Knee |
| 2 | (total knee replacement or total knee arthroplast* or TKR or TKA or TKJR or TKJA).ti |
| 3 | 1 or 2 |
| 4 | *risk/ or exp *protective factors/ or exp *risk assessment/ or exp *risk factors/ |
| 5 | (risk factor* or risk assessment or body mass index or age or gender or comorbidit*).ab. |
| 6 | Exp Body Mass Index/ |
| 7 | Exp *COMORBIDITY/ |
| 8 | 4 or 5 or 6 or 7 |
| 9 | Exp *”length of stay”/ or exp *patient readmission/ |
| 10 | (readmission or length of stay).ti. Or 30-day readmission.kw. or readmi*.ti |
| 11 | (“30” adj4 day).mp. [mp=title, abstract, original title, name of substance word, subject heading word, floating sub-heading word, keyword heading word, protocol supplementary concept word, rare disease supplementary concept word, unique identifier, synonyms] |
| 12 | (thirty adj4 day).mp. [mp=title, abstract, original title, name of substance word, subject heading word, floating sub-heading word, keyword heading word, protocol supplementary concept word, rare disease supplementary concept word, unique identifier, synonyms] |
| 13 | 9 or 10 or 11 or 12 |
| 14 | 3 and 8 and 13 |

| **EMBASE (Ovid) Search Strategy** | |
| --- | --- |
| 1 | Exp *total knee arthroplasty/ |
| 2 | (total knee replacement or total knee arthroplast* or TKR or TKA or TKJA or TKJA.ti. |
| 3 | 1 or 2 |
| 4 | Exp *risk factor/ |
| 5 | (risk factor or risk assessment or body mass index or age or gender or comorbidit*.ab. |
| 6 | Exp *body mass/ |
| 7 | Exp *comorbidity/ or exp *Charlson Comorbidity Index/ or exp *Elixhauser comorbidity index/ or exp *comorbidity assessment/ |
| 8 | 4 or 5 or 6 or 7 |
| 9 | Exp *hospital readmission/ or exp *”length of stay”/ |
| 10 | (readmission or length of stay).ti. or 30-day readmission.kw. or readmi*.ti. |
| 11 | (“30” adj4 day).mp. [mp=title, abstract, heading word, drug trade name, original title, device manufacturer, drug manufacturer, device trade name, keyword, floating subheading word, candidate term word] |
| 12 | (thirty adj4 day).mp. [mp=title, abstract, heading word, drug trade name, original title, device manufacturer, drug manufacturer, device trade name, keyword, floating subheading word, candidate term word] |
| 13 | 9 or 10 or 11 or 12 |
| 14 | 3 and 8 and 13 |
| 15 | Exp time factor/ |
| 16 | 3 and 15 |
| 17 | 14 or 16 |
| 18 | Limit 17 to embase |
